# Phage Therapy in the Management of Urinary Tract Infections: A Comprehensive Systematic Review

**DOI:** 10.1089/phage.2023.0024

**Published:** 2023-09-20

**Authors:** Amany M. Al-Anany, Payton B. Hooey, Jonathan D. Cook, Lori L. Burrows, Julia Martyniuk, Alexander P. Hynes, Greg J. German

**Affiliations:** ^1^ Department of Biochemistry and Biomedical Sciences, McMaster University, Hamilton, Canada.; ^2^ Department of Laboratory Medicine and Pathobiology, University of Toronto, Toronto, Canada.; ^3^ Gerstein Science Information Centre, University of Toronto, Toronto, Canada.; ^4^ Department of Medicine, McMaster University, Hamilton, Canada.; ^5^ Unity Health Toronto, St. Joseph's Health Centre Chronic Infection/Phage Therapy Clinic, Toronto, Canada.

**Keywords:** bacteriophage, phage therapy, urinary tract infection, UTI, kidney transplant, cystitis, bacteriuria, renal transplant, systematic review

## Abstract

Urinary tract infections (UTIs) are a problem worldwide, affecting almost half a billion people each year. Increasing antibiotic resistance and limited therapeutic options have led to the exploration of alternative therapies for UTIs, including bacteriophage (phage) therapy. This systematic review aims at evaluating the efficacy of phage therapy in treating UTIs. We employed a comprehensive search strategy for any language, any animal, and any publication date. A total of 55 *in vivo* and clinical studies were included. Of the studies, 22% were published in a non-English language, 32.7% were before the year 1996, and the rest were after 2005. The results of this review suggest that phage therapy for UTIs can be effective; more than 72% of the included articles reported microbiological and clinical improvements. On the other hand, only 5 randomized controlled trials have been completed, and case reports and case series information were frequently incomplete for analysis. Overall, this comprehensive systematic review identifies preliminary evidence supporting the potential of phage therapy as a safe and viable option for the treatment of UTIs.

## Introduction

Urinary tract infections (UTIs) are a global health concern, contributing to 404.6 million cases annually in both males and females, with 236,786 deaths.^1^ Approximately 50% of women will get a UTI at least once in their lifetime, and of those, 20–30% will have a recurrence.^2^ Consequently, UTIs act as a driver for the development of antimicrobial resistance (AMR), affecting quality of life and creating a massive clinical and economic burden.^3^

Threats from superbugs and AMR associated with UTIs have rekindled interest in viruses that exclusively target bacteria (bacteriophages, commonly known as phages).^4^ A century after their discovery, bacteriophages are now receiving attention as a promising solution to AMR. First, bacteriophages interact specifically with their bacterial host and have a narrow spectrum of activity.

Accordingly, compared with antibiotics, phages cause less harm to patients' microbiota.^5^ While the interaction specificity is challenging in cases of polymicrobial infections, as they would require administering more than one phage, this is not a hurdle in UTIs as they are not primarily polymicrobial in nature at time of diagnosis.^6^ Second, phage therapy is characterized by auto-dosing, as phages are self-replicating at the site of infection as long as susceptible target bacteria are nearby.^7^ Third, there is evidence that phages are more effective than antibiotics in the treatment of biofilms associated with UTIs,^8–10^ which are important contributors to antibiotic treatment failure and recurrence.^3,11^

Between the 1940s and 1970s, lack of widespread knowledge about phages in combination with the rise of the golden age of antibiotic discovery led to the abandonment of phage therapy in Western Europe and in the United States. However, Eastern Europe and the former Soviet republics continued experimenting with phages and using them for therapy.^12^

In the past three decades, the prevalence of antibiotic-resistant superbugs and the lack of novel classes in the antibiotic pipeline has led to a revival of phage therapy globally, with multiple centres now building on the long-standing experience from Poland and the former Soviet Union.^13^ These centers, in addition to regulatory bodies, are looking for animal studies (preclinical) to support phage clinical trials in humans. So, a targeted systematic review on phage therapy in urinary infections in humans and laboratory animals will assist further study and phage therapy protocol development.

Four phage therapy narrative reviews and a systematic review with a small section on UTI management have been published (Table 1), but these are limited in scope with respect to timeframe, language, and/or research subjects.^14–18^ Thus, we sought to generate a comprehensive systematic review that includes both a One Health lens and non-English language publications on the role of phages as a suitable alternative or adjuvant therapy for managing UTIs in humans and animals.

**Table 1.** Literature Reviews Covering Bacteriophage Therapy for Urinary Tract Infections

Review title	Type	Publication year	Limitation	References
Managing urinary tract infections through phage therapy: A novel approach.	A narrative review	2019	Focuses on UPECs and primarily in vitro studies.	^15^
Prospects of using bacteriophages in urological practice.	A narrative review	2019	Non-English language: Russian, which results in difficulty in data extraction and interpretation.	^16^
Phage prevalence in the human urinary tract—Current knowledge and therapeutic implications.	A narrative review	2020	Focuses on phage presence within the urobiome and its possible implications for health and disease.	^18^
Bacteriophages: A panacea in neuro-urology?	A limited narrative review	2020	A short review that focuses on UTIs in patients with neurogenic lower urinary tract dysfunction.	^14^
Safety and efficacy of phage therapy in difficult-to-treat infections: A systematic review.	A systematic review in 2022 contained a section on UTIs	2022	Limited to articles starting from 2000, English literature only, and studies only in humans.	^17^

UPEC, uropathogenic *Escherichia coli*; UTI, urinary tract infection.

A systematic review was conducted in conformity with the Preferred Reporting Items for Systematic Reviews and Meta-Analyses (PRISMA) guidelines (summarized in Supplementary Table S1) and following Cochrane methodology.^19^ First, a preliminary search was conducted on November 20, 2022, in ERO, PubMed, and the Cochrane library to identify overlap among published and registered systematic reviews (summarized in Table 1).

## Methods

### Search methods

An information specialist and health science librarian (co-author J.M.) in consultation with the review team identified peer-reviewed literature using a comprehensive search strategy. The PRISMA-Search extension developed by Rethlefsen et al.^19a^ has been used to report all search strategies. To minimize search errors and enhance comprehensiveness, a health science librarian peer-reviewed the search strategy following the Peer Review of Electronic Search Strategies (PRESS) form.^20^ Relevant subject headings and text words related to the following concepts were included in the search: “phage” therapy and “UTI.” For comprehensiveness, no date, language, or design limits were applied.

A protocol was registered in PROSPERO database (ID CRD4202231927); the search strategy was developed and finalized in OVID Medline, and then translated to OVID Embase, EBSCO CINAHL, the Cochrane Library, Scopus, and Global Index Medicus.

The search was run on March 18, 2022, and rerun on November 17, 2022. The articles were exported into Endnote, de-duplicated, and then imported into Covidence (Covidence systematic review software, Veritas Health Innovation, Melbourne, Australia. Available at www.covidence.org) for screening. The full search strategies can be viewed here: https://doi.org/10.5683/SP3/WIVREO

Gray literature was identified by manually searching relevant gray literature databases, catalogues, and search engines, including ClinicalTrials.gov, WHO International Clinical Trials Registry Platform, Open Grey, TripPro, and OSIster. The gray literature search documentation can be located here: https://doi.org/10.5683/SP3/WIVREO

The reference lists of included articles were also searched. In September and October 2022, P.B.H. conducted a supplementary search of each primary article's references and citing articles to obtain studies missing from the initial search. Reference searching was conducted initially using Spidercite (Available at https://sr-accelerator.com/#/spidercite), while not possible, Web of Science and Scopus were used for the remaining articles.^21,22^

On November 18, 2022, the search results were input directly into Covidence and automatically deduplicated. Next, the articles underwent title and abstract screening and a full-text review as described earlier. A total of six results from the supplementary search were advanced to the data extraction stage.

### Study selection (inclusion and exclusion criteria)

Before the title-abstract and full-text screening processes (A.M.A., P.B.H., and G.J.G.) performed a pilot test with at least 10% of the articles to ensure reviewers were mostly in agreement regarding the eligibility criteria. The initial round of title and abstract screening was performed by two independent reviewers (A.M.A. and P.B.H.), with a third reviewer (G.J.G.) resolving disagreements. Full manuscripts were next obtained and uploaded to a shared cloud-based repository for access by all reviewers. Articles that were published in languages other than English were converted from PDF to Word using the Optical Character Recognition (OCR) program by Convertio (see https://convertio.co/) and translated using Google Translate (available at https://translate.google.ca/).^23^

**Fig. 1.**
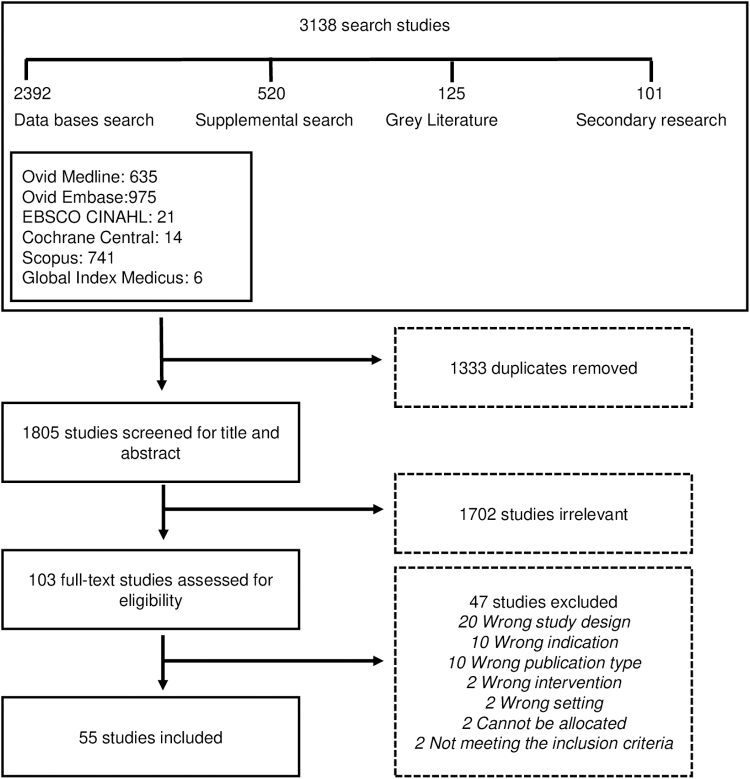
Systematic review characteristics at a glance.

EurekaMag (see https://eurekamag.com/), a scholarly article and abstract supply service, was used to access five articles. Articles were assessed as eligible based on the study design, the condition being studied, population, intervention, comparator, and outcomes (Fig. 1). After all articles from the initial search were obtained, eligibility screening of the full-text manuscripts was conducted with two independent reviewers (P.B.H. and A.M.A. or G.J.G.). A third reviewer (A.P.H.) was the adjudicator on the eligibility and the reason for exclusion.

### Data extraction

One reviewer (P.B.H.) completed data extraction using Google Sheets, and each of the extractions was validated by another reviewer (A.M.A.). In cases of missing information, Supplementary Material was reviewed, and efforts were made to contact the authors. A third reviewer (A.P.H.) was consulted on areas of disagreement.

Data retrieved from the selected studies included: language, method of study, participant's demographics (age, gender, and type of animal or animal model), baseline characteristics (organism of infection, type of infection, and previous treatments), study design/regimen (selected phage(s), phage origin or isolation source, nucleic acid sequence GenBank number, route, concentration, timing, dosing, frequency, and additional therapeutics), and measures of effect (microbiological outcomes, clinical outcomes, and presence of phage resistance).

### Critical appraisal

The Joanna Briggs Institute Critical Appraisal Checklist was used to evaluate case series and case reports.^24^ For case reports and case series to be accepted, they needed to receive at least five categories with “Yes” or “Unclear.” Critical appraisal of the human randomized control trials (RCTs) was performed using the Critical Appraisal Skills Program (CASP) Checklist.^25,26^ Human RCTs needed to meet the threshold of at least eight categories with “Yes” or “Unclear” to be accepted. If a study did not meet these thresholds during the critical appraisal, then they were marginally accepted. Each study was evaluated independently by two reviewers (J.D.C. and P.B.H.). Discrepancies in the decision to include or exclude studies were reviewed and resolved by an additional reviewer (G.J.G.).

## Results

### Study characteristics

**FIG. 2.**
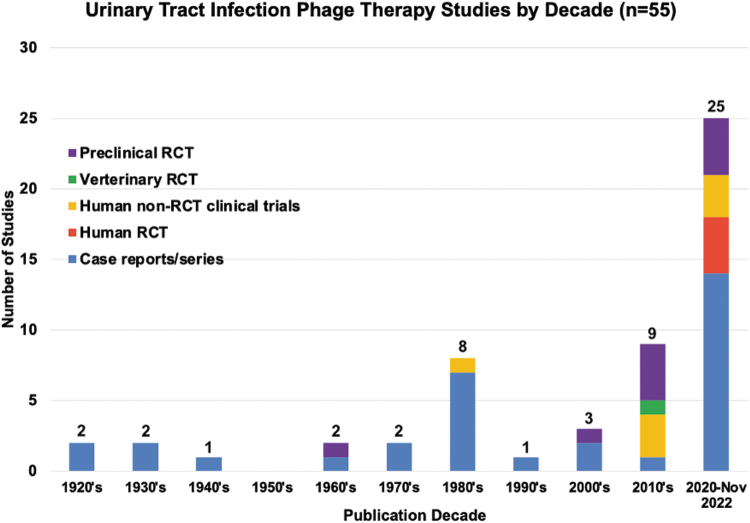
PRISMA flow chart summarizing the study selection process. PRISMA, Preferred Reporting Items for Systematic Reviews and Meta-Analyses.

The initial data search returned 3138 search results, leaving 1805 manuscripts to be screened after de-duplication for title and abstract. Following this, 103 studies were assessed for eligibility; 55 articles fulfilled the selection criteria and were included in the systematic review (Fig. 2). Most studies were published in English (*n* = 43) or Russian (*n* = 8).

**FIG. 3.**
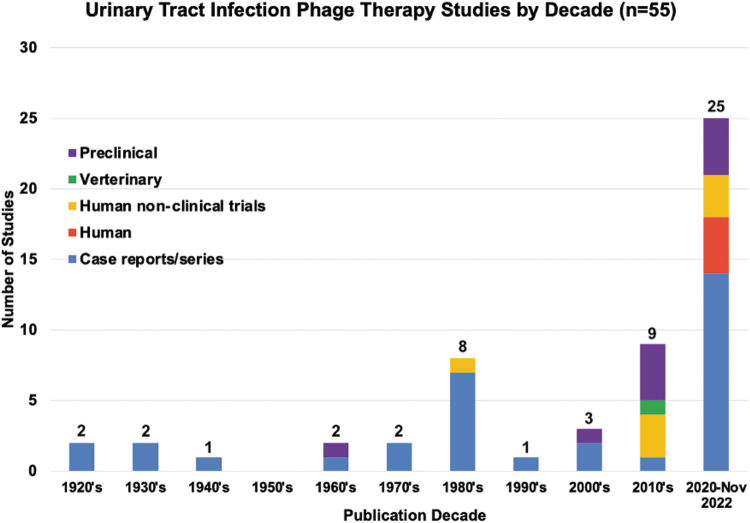
Urinary tract infection phage therapy studies by decade (*n* = 55). RCT, randomized control trial; Preclinical, studies designed to evaluate the effectiveness of a treatment for an animal model; Veterinary RCT, RCT designed to evaluate the effectiveness of an intervention for an animal; Human non-RCT clinical trial, human trial that does not include an element of randomization; Case report, report of the symptoms, diagnosis, treatment, and follow-up of an individual patient; Case series, report of the symptoms, diagnosis, treatment, and follow-up of several patients.

Two articles were published in French and two in Mandarin. Regarding the method of study, 34.5% (*n* = 19/55) were case series, 25.4% (*n* = 14/55) were case reports, 7.2% (*n* = 4/55) were RCTs, 12.7% (*n* = 7/55) were non-randomized control clinical trials (non-RCT clinical trial), and 20.0% (*n* = 11/55) were preclinical studies (Table 2). Included articles were published between 1926 and 2022 (Fig. 3), 37 articles were published after 2005, and the rest (18/55) were published before 1996.

**Table 2.** Studies Assessing the Safety and Efficacy of Phage Therapy in Managing Urinary Tract Infections (27–81)

Author, date, Ref. and language	Method of study	Host demographics	Infectious species	Route of phage administration	Dose of phage	Frequency of phage administration	Duration of phage administration	Antibiotic use	Microbiological improvement	Clinical improvement	Follow up length
Larkum (1926)^50^ (English)	Case series	Female children and adults (*n* = 4)	*Escherichia coli* or *Colon bacillus*	Renal pelvis or bladder installation, subcutaneous inoculation, or NS	2, 5, 10 cm^3^ or NS	1 Time daily or NS	1–2 Days or NS	NS	Yes	Yes	Several weeks or up to 2 years
Caldwell (1928)^39^ (English)	Case series	Human (*n* = 12)	*E. coli* *B. pyocyaneus* *Atypical B. pyocyaneus*	Subcutaneous injections and lavage of the bladder	NS	Usually 1 time daily or every other day	3–6 Injections	NS	Yes	No	At least 1 month
Schultz (1932)^29^ (English)	Case series	Human (*n* = 151)	*E. coli*	NS	NS	NS	NS	NS	Yes	Yes	At least 3 months
Wehrbein and Nerb (1935)^77^ (English)	Case series	57-Year-old male and a 37-Year-old female	*E. coli* or NS	Kidney installation	NS	1–2 Times daily	2, 5, or 7 days apart	No	NS	Yes	At least 6–8 days
Pasricha and DeMonte (1941)^60^ (English)	Case report	52-Year-old male	*Bacterium alkalescens*	Oral	2 cm^3^ in 2 oz of water	3 Times daily	1 Month	No	No	No	NS
Bertoye and Courtier (1960)^28^ (French)	Case report	NS	*Pyocyanin bacilli*	Cystostomy	NS	NS	NS	Yes	NS	Yes	NS
Manolov and Kosturkov (1965)^57^ (Russian)	Preclinical	Guinea Pigs (*n* = 27, 3 groups)	*Shingella flexneri*	Bladder injection	1 mL	1 Time	1 Day	No	NS	No	At least 8 days
Zilisteanu et al. (1971)^80^ (French)	Case series	Human (*n* = 56, 4 groups)	*Citrobacter*, *Pseudomonas pyocyanea* and/or *E. coli*	Oral	20 mL	1 Time daily	5 Days	Yes (for 2 groups only)	Yes	NS	At least 10–12 months
Proskurov (1973)^62^ (Russian)	Case report	32-Year-old male^a^	*Staphylococcus aureus*	Injection into paraprostatic space	5 mL	1 Time daily	6 Days	No	Yes	Yes	2 Years
Slopek et al. (1983)^30^ (English)	Case series	Male and female children and adults (*n* = 7)	NS	Oral and bladder lavage	10 mL or NS	3 Times daily or NS	1.5–4 Weeks	Yes (for 1 patient only)	Yes	Yes	NS
Slopek et al. (1983)^66^ (English)	Case series	Human (*n* = 9)	*Staphylococcus, Klebsiella, E. coli, Proteus*, or *Pseudomonas*	Orally with or without local irrigation of bladder or fistula	10 mL	3 Times daily (Slopek et al., 1983)	1.5–19 Weeks	NS	NS	Yes	NS
Slopek et al. (1984)^67^ (English)	Case series	Male and females (*n* = 11)	*Pseudomona*s, *Staphylococcus*, *Klebsiella*, *Proteus*, and/or *E. coli*	NS	NS	NS	NS	NS	Yes	Yes	NS
Slopek et al. (1985)^68^ (English)	Case series	Male and females (*n* = 31)	*Staphylococcus*, *Pseudomonas*, *E. coli*, *Klebsiella*, and/or *Proteus*	Oral or topical	10 mL or NS	3 Times daily or NS	NS	NS	Yes	Yes	NS
Slopek et al. (1985)^69^ (English)	Case series	Male and female children and young adults (*n* = 8)	*Staphylococcus*, *E. coli*, and/or *Pseudomonas*	Oral and bladder installation	10 mL or NS	3 Times daily or NS	NS	NS	Yes	Yes	NS
Slopek et al. (1985)^70^ (English)	Case series	Male and females (*n* = 14)	*Staphylococcus*	Oral and bladder installation	10 mL or NS	3 Times daily or NS	NS	NS	Yes	Yes	NS
Slopek et al. (1987)^71^ (English)	Case series	Male and female patients (*n* = 42)	*Pseudomonas*, *Staphylococcus*, *Klebsiella*, *Proteus*, and/or *E. coli*	NS	NS	NS	NS	NS	Yes	Yes	NS
Weber-Dabrowska et al. (1987)^76^ (English)	Preclinical	Human (*n* = 40)	*Staphylococcus*, *Klebsiella*, *E. coli*, *Proteus*, or *Pseudomonas*	Oral	20 mL administered to ÿontro ÿontrol, likely same dose for patients	NS	10 Days	NS	Yes	NS	NS
Perepanova et al. (1995)^61^ (Russian)	Case series	Human (*n* = 46)	*Pseudomonas aeruginosa*, *E. coli*, *Proteus mirabilis*, and *Proteus vulgaris*	Topical into the bladder, wound area, into the renal pelvis and oral	5–7, 10–20, 50, or 100 mL	1–2 Times daily	7–10 Days (2 rounds)	No	Yes	Yes	2–3 Weeks
Leszczynski et al. (2006)^52^ (English)	Case report	30-Year-old human	*S. aureus*	Oral	10 mL	3 Times daily	4 Weeks	No	Yes	NS	6 Months
Nishikawa et al.(2008) ^58^ (English)	Preclinical RCT	6–8-Week-old female mice (*n* = 44, 6 groups)	*E. coli*	Intraperitoneal	1 mL	1 Time	1 Day	No	NS	Yes	At least 7 days
Letkiewicz et al. (2009)^55^ (English)	Case series	Males (*n* = 3)	*Enterococcus faecalis*	Rectal	10 mL	2 Times daily	28–33 Days	No	Yes	Yes	8–25 Weeks
Letkiewicz et al. (2010)^54^ (English)	Case series	Males (*n* = 22)	*E. faecalis*, *E. coli*, *Klebsiella pneumoniae*, *P. aeruginosa*, and *S. haemoliticus*	Rectally, orally and/or topically to the glans penis	NS	NS	22–99 Days	NS	Yes	Yes	NS
Khawaldeh et al. (2011)^47^ (English)	Case report	67-Year-old woman	*P. aeruginosa*	Bladder installation	20 mL	1 Time every 12 h	10 Days	Yes	Yes	Yes	1 Year
Tothova et al. (2011)^72^ (English)	Preclinical	10–11-Week-old female mice (*n* = 14)	*Cronobacter turicensis*	Intraperitoneal	100 μL	1 Time daily	1 Day	No	Noᵇ	Yes	24 H
Pagava et al. (2012)^59^ (Russian)	Non-RCT clinical trial	Children (*n* = 7)	NS	Oral	NS	NS	NS	NS	NS	Noᵇ	NS
Zorkin and Shakhnovsky (2013)^81^ (Russian)	Non-RCT clinical trial	Children (*n* = 166, 2 groups)	*E. coli*, *Proteus, Klebsiella*, *P. aeruginosa*, *Enterobacter*, *Serratia*, and *Enterococcus*	Oral and local irrigation of the pelvicalyceal cavity, bladder cavity, or lumen of the ureter	5–10 or 20–30 mL	3 Times daily or NS	5–7 Days	Yes	Yes	Yes	6–12 Months
Deng et al. (2016)^42^ (Mandarin)	Preclinical	8–12-Week-old BALB/c mice (*n* = 12)	*Acinetobacter baumannii*	NS	1 mL	1 Time daily	7 Days	No	NS	Yes	7 Days
Dolgolikova et al. (2020)^48^ (English)	RCT	Human females (*n* = 30, 2 groups)	*E. coli, K. pneumoniae or Enterobacter* spp.	NS	20 mL	2 Times daily	10–14 Days	Yes	NS	Yes	At least 6 months
Dufour et al. (2016)^43^ (English)	Preclinical	Mice (*n* = 23)	*E. coli* and *Colo coli*	Intraperitoneal	NS	1 Time daily	1 Day	NS	Yes	NS	At least 48 h
Ujmajuridze et al. (2016)^74^ (English)	Non-RCT clinical trial	Human (*n* = 9)	*E. coli*, *Enterococcus*, *Pseudomonas*, or *Streptococcus*	NS	NS	NS	NS	NS	Yes	NS	NS
Chen et al. (2018)^40^ (English)	Preclinical	5-Week-old female mice (*n* = 18, 3 groups)	*Pasteurella multocida*	Intraperitoneal	100 μL	1–2 Times daily	1 or 5 days	No	NS	Yes	21 Days
Luo et al. (2018)^56^ (English)	Veterinary RCT	Nile tilapia (*n* = 30)	*Streptococcus agalactiae*	Intraperitoneal	NS	NS	NS	No	Yes	Yes	14 Days
Ujmajuridze et al. (2018)^73^ (English)	Non-RCT clinical trial	Males (*n* = 9)	*E. coli*, *Streptococcus*, *Enterococcus*, or *Pseudomonas*	Bladder installation	20 mL	Every 12 h	3 or 7 Days	Yes (for 1 group only)	Yes	NS	NS
Aslanov and Dolgiy (2020)^36^ (English)	Case series	Human (*n* = 60)	*P. aeruginosa*, *Proteus* spp. and *E. coli*	Bladder installation	NS	NS	1–5 Days	NS	Yes	Yes	NS
Bao et al. (2020)^37^ (English)	Preclinical	6–8-Week-old female mice (*n* = 80, 6 groups)	*S. enteritidis*	Intraperitoneal	200 μL or NS	1 Time daily	1 Day	No	No	Yes	10 Days
Bao et al. (2020)^38^ (English)	Case report	63-Year-old female	*K. pneumoniae*	Bladder irrigation	50 mL or NS	1 Time daily or NS	5 Days (3 rounds)	Yes (for 1 round only)	Yes	Yes	6 Months
Corbellino et al. (2020)^41^ (English)	Case report	57-Year-old female	*K. pneumoniae*	Oral and intrarectal	10 mL oral or 10^6^ suppository	1 Time every 12 h	2 or 3 Weeks	No	Yes	Yes	11 Months
Kuipers et al. (2019)^49^ (English)	Case report	58-Year-old male	*K. pneumoniae*	Oral and bladder irrigation	NS	1–2 Times daily or 1 time every other day	2 or 8 Weeks	Yes	Yes	Yes	14 Months
Qin et al. (2021)^64^ (English)	Case report	66-Year-old male	*K. pneumoniae*	Bladder irrigation and kidney irrigation	10 and/or 50 mL	Every 48 h	2 Weeks (4 rounds)	Yes (for 1 round only)	Yes	Yes	2 Months
Vasilyev et al. (2020)^75^ (Russian)	Non-RCT clinical trial	Females (*n* = 73)	Predominantly *E. coli*	Intraurethral injection	10 mL	NS	NS	No	Yes	Yes	10 Days
Zeng et al. (2020)^79^ (Mandarin)	Case report	65-Year old female	*K. pneumoniae*	Continuous bladder infusion	200 mL	1 Time daily	5 Days (2 rounds)	Yes (for 1 round only)	Yes	Yes	6.5 Months
Ali et al. (2021)^35^ (English)	Preclinical	Female mice (*n* = 150, 5 groups)	*E. coli*	Intraperitonal and/or transurethral	100 μL	1 Time daily	1 Day	No	Yes	NS	10 Days
Govorov et al. (2021)^45^ (English)	Non-RCT clinical trial	Humans (*n* = 300)	NS	Intraurethral injection of phage-based gel	10 mL	NS	NS	No	Yes	Yes	3 Days
Johri et al. (2021)^46^ (English)	Case report	33-year-old male	*S. haemolyticus*, *S. aureus*, *E. faecalis*, and *S. epidermidis*	Oral, rectal suppositories, and urethral instillations	10 mL, 20 mL or NS	2 Times per day or NS	14 Days or 2–4 months (3 rounds)	No	Yes	Yes	At least 4 months
Kim et al. (2021)^27^ (English)	RCT	Human (*n* < 30)	*E. coli*	NS	NS	NS	NS	NS	Yes	NS	At least 24 h
Leitner et al. (2020)^51^	RCT	Males (*n* = 28)	*Enterococcus* spp., *E. coli*, *Proteus mirabilis*, *P. aeruginosa*, *S.* spp., and *Streptococcus* spp.	Intravesical	20 mL	2 Times daily	7 Days	NS	Yes	Yes	7 Days
Letkiewicz et al. (2021)^53^ (English)	Case series	Human (*n* = 15)	*E. coli*, *K. variicola*, *K. pneumoniae*, *E. Fecalis*, and/or *P. aeruginosa*	Intravesical and intravaginal	10 mL	2 Times daily	3 Days (1–2 rounds)	NS	Yes	Yes	NS
Rostkowska et al. (2020)^65^ (English)	Case report	60-Year-old male	*K. pneumoniae*	Intra-rectal	10 mL	2 Times daily	29 Days	Yes	Yes	Yes	Approximately 4 years
Terwilliger et al. (2021)^32^ (English)	Case report	56-Year-old male	*E. coli*	Intravenous and peripherally inserted central catheter	10^9^ pfu/mL	Every 12 h	2 Weeks	Yes	Yes	Yes	12 Weeks
Zaitsev et al. (2021)^33^ (Russian)	RCT	Females (*n* = 75, 3 groups)	*E. coli*, *Klebsiella* spp., *E. faecalis* and *Proteus mirabilis*	Bladder installation and intra-rectal	50 mL or NS	1–2 Times weekly	12 Weeks	NS	Yes	Yes	90 Days
Zaldastanishvili et al. (2021)^78^ (English)	Case series	72-Year-old female	*K. pneumoniae*	Oral and vaginal suppository	NS	1–2 Times daily or NS	10, 15, or 20 Days (4 rounds)	Yes	No	No	Approximately 1 month
Abed et al. (2022)^34^ (English)	Preclinical	Female albino mice (*n* = 60, 2 groups)	*E. coli*	Transurethral and intraperitoneal	100 μL	1 Time daily	1 Day	No	Yes	NS	10 Days
Gainey et al. (2022^31^ (English)	Case report	17-Year-old female	*E. coli*	Intravenously	NS	1 Time per day	21 Days	Yes	Yes	Yes	4 Years
Gordillo Altamirano et al. (2022)^44^ (English)	Preclinical	6–10-week-old mice (*n* = 26, 6 groups)	*A. baumannii* AB9a	Intraperitoneal	5 × 10^6^ pfu (MOI = 1)	1–2 Times per day	1 Day	Yes (for 2 groups only)	Yes	NS	11–16 H
Pushkar et al. (2022)^63^ (Russian)	Case series	Human (*n* = 3)	*E. coli* or *K. pneumoniae*	Intravesical installation	50 mL	2 Times weekly	12 Weeks	NS	Yes	Yes	NS

See Supplementary Table S2 for the comprehensive data extraction sheet.

^a^ A total of 12 patients received phage therapy, but little detail was provided.

^b^ Microbiological or clinical improvements were observed, but statistical significance was not achieved.

MOI, multiplicity of infection; NS, not specified in the study.

### Human/animal subject characteristics

Of the studies, 14.6% were performed on mice (*n* = 2703), with the rest as follows: 1.82% in guinea pigs (*n* = 37), 1.82% in Nile tilapia (*n* = 120), and 81.8% were performed in humans (*n* = at least 2083), where “n” is the actual number of subjects involved. One article^27^ published in 2021 mentioned that the number of included patients was “<30,” whereas three other studies published in 1932, 1960, and 1983^28–30^ failed to specify the number of patients.

Most infections, accounting for 38.2% (21/55) of the included studies, were caused by *Escherichia coli*, as either a mono- or polymicrobial infection. Other pathogens included *Acinetobacter baumannii*, *Citrobacter* spp., *Cronobacter turicensis*, *Enterococcus faecalis*, *Klebsiella pneumoniae*, *Salmonella enteritidis*, *Staphylococcus aureus*, *Shigella flexneri*, *Pseudomonas aeruginosa*, *Proteus mirabilis*, and *Proteus vulgaris*.

There was a single study that failed to specify the infectious organism. Patients were treated previously with antibiotics (*n* = 18/55), surgeries (*n* = 5/55), but in 17 articles previous treatments were not specified.

### Phage characteristics

Among the 55 articles included, 13 reported using a single phage, 4 studies used 2 phages, and 9 studies used 3 or more phages. The remaining 29 articles did not specify the number of phages used. In eight studies, phages were purified by ultracentrifugation, one study by filter sterilization, one by anion exchange concentration and then dialyzed followed by filter sterilization. Only seven articles specified accession numbers for the phage genome in GenBank or other repositories. Phages were used at concentrations from 10^4^ plaque-forming units per mL (PFU/mL) up to 3 × 10^11^ PFU/mL.

Phages were introduced via different routes, with 36.3% (20/55) using an intravesicular approach such as bladder instillation, bladder injection, bladder irrigation, or bladder lavage. Phages were given via a single route or through a combination of different routes, including intraperitoneally, subcutaneously, kidney instillation, transurethrally, intrarectally, and orally.

Only in 3.6% of the articles (2/55) were phages given intravenously, and both were recent case reports.^31,32^ In one study, bladder instillation was compared with intrarectal or both routes of administration concurrently.^33^ Phages were also given topically, via cystostomy, and intravesically with or without vaginal administration.

Nine studies failed to specify the route of administration. Phages were given at various frequencies; once, twice, or three times daily; once every 2 days, once every week, or two times weekly. Frequency of phage administration was not identified in 17 trials, and the duration of phage administration ranged from 1 day to 19 weeks.

In many trials, phages were given in addition to other therapeutics, including toxoids, autovaccines, anesthetics, surgical treatments, the herbal product *Centella asiatica*, phage encoded polymerases as depolymerase Dep-ORF8, and antibiotics.

The antibiotics administered included ceftazidime, meropenem, imipenem/amikacin, imipenem/cilastatin, amikacin, colistin (polymyxin E), polymyxin B, fosfomycin, third-generation cephalosporin; ceftriaxone, ertapenem, fluoroquinolones, ciprofloxacin, neomycin, colimycin, trimethoprim-sulfamethoxazole (SMZ-TMP), piperacillin-tazobactam, ceftriaxone, and amoxicillin/clavulanic acid. In 11 RCTs, 4 case reports, 3 case series, and 2 clinical trials, no additional therapeutics were given with phages.

### Efficacy of phage therapy

In most articles, phage administration resulted in microbiological and clinical improvements (78.2% (43/55) and 72.7% (40/55), respectively). In terms of microbiological effectiveness, 62% of the articles (34/55) reported complete bacterial eradication, urine sterilization, bacterial levels below the limit of detection, or a negative result for culture growth.

The treatments that resulted in eradication included 75.5% (670/892) of human patients and 71.4% (150/210) of mice. Moreover, only 0.8% of those treated experienced re-infection after 2–4 months, 4.7% after 10–12 months, 0.1% after 2–7 weeks post-treatment, and 1.8% experienced a recurrence of infection without a defined timeframe. In a single case report, a new bacterial variant evolved that was sensitive to phage administration.^32^

A decrease in bacterial load was reported in 29% of the articles (16/55); these included 35% of mice (66/189), 50% of Nile tilapia (30/60), and 42.7% (207/485) of human patients. Interestingly, in two preclinical studies, a decrease in bacterial load was reported for one group of mice and not for the other. In the first article, authors performed two stage preclinical study comparing four groups of mice.

These groups were treated with either phosphate buffered saline, antibiotic, phage, or combination of phage + antibiotic. In this first stage, bacterial loads decreased in both phage and phage + antibiotic groups. This was then followed by a second stage where only phage groups were compared with phage + antibiotic group. In this second stage, bacterial loads were significantly lower in phage + antibiotic group compared with phage-only group.^82^

In the second article, only the group of mice that were given high concentrations of phage showed microbiological efficacy.^37^ In one of the reported cases described in a case series, a single female patient experienced an increase in bacterial load after the first course of treatment; however, the bacterial load was not assessed at the end of the second course of phage therapy.^53^

Improvement of clinical symptoms was reported in most human patients; 97% (>995/1026), with relapse in just over 1.7% (17/1026). It is worth noting that in the 2010 Letkiewicz trial that included 22 patients, all but 2 cases experienced substantial clinical improvements.^53^ One was reported as “not possible to evaluate” whereas in the second patient, no change in intensity of symptoms after treatment was reported.^53^ Another study reported significant improvements with a relapse afterward, without specifying the number of enrolled subjects.^28^

In a single clinical trial, clinical improvements were observed, but statistical significance was not achieved.^59^ In only one of the 55 studies, a 72-year-old female patient found phage therapy unhelpful.^78^ A table containing only human subjects stratified by organisms reported, therapy type, route of administration, efficacy, and safety can be found in Table 3.

**Table 3.** Human Subjects Sub-Group Results on Phage Therapy for Urinary Tract Infections

Organism		Percent (specified)		
	Gram negative (%)	88.90		
	Gram positive (%)	3.40		
	Gram negative and Gram positive (%)	7.60		

Therapy		Total	Percent	
	Phage alone	557	39.12	
	Phage+Abx^a^	261	18.33	
	Not specified	606	42.56	
	Total	1424		

Route of administration		Total	Percent	
	PO	343	23.92	
	Parenteral	11	0.77	
	Intravesicular	579	40.38	
	Topical/rectal	30	2.09	
	Combination	205	14.30	
	Not specified	266	18.55	
	Total	1434		

Efficacy		Total	Percent (specified)	Percent (total)
	Marked clinical improvement/cure	741	78.91	51.10
	Clinical improvement	145	15.44	10.00
	No clinical improvement	53	5.64	3.66
	Clinical improvement not specified	511		35.24
	Total when specified	939		
	Total	1450		
	Marked microbiologic improvement/eradication	726	66.67	63.52
	Microbiological improvement	149	13.68	13.04
	No microbiological improvement	214	19.65	18.72
	Microbiological improvement not specified	54		4.72
	Total when specified	1089		
	Total	1143		

Safety			Percent (specified)	Percent (total)
	No adverse events	621	98.73	43.37
	Adverse events reported	8	1.27	0.56
	Adverse events not specified	803		56.08
	Total when specified	629		
	Total	1432		

Tabulated data enumerating results from human case reports, case series, and clinical trials throughout the review period (until November 17, 2022). See Supplementary Table S5 for data extraction sheet.

^a^ Abx can include vaccine or toxoid therapy.

PO, oral administration.

In terms of phage resistance, 80% of the articles failed to specify its incidence. Two studies (3.6%) stated that no phage resistance was observed, whereas in the remaining studies (16.1% *n* = 9) phage resistance was reported. In the largest case series (*n* = 25) that reported the development of resistance, 21% of 90 isolates recovered following treatment had become resistant to phages. A further 4.3% were resistant to both the phages and antibiotics that were used for treatment.^80^ In all instances, expanded phage susceptibility testing was not reported.

### Safety of phage therapy

In most of the reported cases, phages were safe, with no reported adverse effects. While 58.1% of the articles failed to specify whether adverse effects were seen, 20 studies (36.3%) reported no adverse effects, and only 3 studies (5.45%) described adverse effects after phage administration. In the first study, one patient experienced a sudden fever and chills on the third day after phage administration, which was attributed to the pre-existing *P. aeruginosa* infection.^73^

In another, the patient initially experienced an increase in testicular and back pain after starting treatment, but those symptoms subsided after three weeks.^46^ Finally, in the third article, 21% of patients treated with phage therapy experienced adverse events: 4% were grade 1, 4% were grade 2, 11% were grades 3, and 4% were lost to follow-up. Of note, this was in comparison to 41% of the placebo group and 30% of the antibiotic group who experienced diverse effects in that same trial.^51^

### Critical appraisal of phage therapy studies

There was a total of 14 case reports included in our review, with 1 case report being marginally accepted following the critical appraisal. A total of 17 case series were assessed, with 6 case series being marginally accepted. Meanwhile, all four human RCTs were included. Critical appraisal summaries for case reports, case series, and human RCTs used in our analysis can be found in Table 3 and Supplementary Table S3 (case reports and case series), and Supplementary Table S4 (Human RCTs).

## Discussion

In the past decade, there has been a resurgence of interest in phages and their potential to overcome antibiotic-resistant and chronic urinary tract-associated infections. This systematic review is intended to provide an overview of the extent, range, and nature of all available UTI phage therapy trials, and to synthesize data to help in the evaluation of phages as an option for control of UTIs. It is the first systematic review to assess phage therapy efficacy in UTIs without restrictions in terms of language or time frame.

We followed Cochrane methodology and PRISMA guidelines, ensuring that the review is comprehensive, reproducible, transparent, and with the goal of being unbiased. Following careful screening, we reviewed 55 articles that investigated the use of phages in humans and in animal models, including case studies, case reports, preclinical, RCTs, and non-randomized control clinical trials.

It is encouraging that more than half of the articles fulfilling our inclusion criteria (65.4%, 37/55) were published in the past 19 years, with a gap in publications from 1996 through 2005 (Fig. 3). This distribution reflects the recent increase in interest and experience in the use of phage therapy for UTIs.

The success rate of phage therapy was defined based on microbiological and clinical outcomes, and >72.7% of the included articles reported improvements following phage administration. However, of the 93 phage therapy studies covered by the 55 articles, all but 17 studies used concomitant treatments. These confounders make it harder to determine the explicit contribution of phages in treatment success.

Our systematic review reports fewer clinical improvements compared with another^17^ that included a section on phage therapy in UTIs. The latter reported 100% clinical improvement in all included articles. This discrepancy may be due to differences in the number of articles included. We placed no restrictions on timeframe and language search, whereas the other systematic review included only English-language studies published between 2000 and 2021.

Analysis on phage therapy UTI data focusing on human subjects in greater detail is revealing. As seen in Table 3 and Supplementary Table S5, out of the roughly 1400 distinct patient applications or study arms (Patients may be used more than once in a subsequent arm), phages were used alone in 39% of cases, with antibiotics in 18% of cases, and not specified in 43% of applications. Moreover, the route of administration had a plurality of intravesicular (urinary bladder and rarely kidney pelvis) routes, with less occurrences of oral, or combination therapy.

Clinical efficacy when specified was 79% for marked improvement/cure, 15% for improvement, and no improvement in 6%; unfortunately, there was a high percentage of non-specified distinct applications, which was 35% of the 1450 study arms. Microbiological outcomes showed marked improvement and improvement in 67% and 14% of cases, respectively.

Of the 1089 evaluated applications or study arms, only on 54 occasions microbiology outcome was not specified. Lastly, no adverse events were indicated in 621 phage therapy patients (99% when specified) and in only eight situations did a patient report a mild adverse event (1% when specified). This was limited due to the high number of situations where adverse events were not specified: 56% over a total of 1432 occurrences.

Importantly, we identified and analyzed four recent articles reporting the results of RCTs using phage therapy to treat UTI, three of which were not included in prior analyses. The Dolgolikova et al. RCT^48^ was a three-armed trial assessing recurrent UTI following kidney transplant. They showed that with standard of care, the average number of UTI recurrence in the following year was seven, in the phage therapy group combined with antibiotics the average yearly UTI recurrence was reduced to two, and in the group that received phage therapy with antibiotics and a probiotic solution, the recurrence rate was zero.

This is in keeping with other studies in this review that show increased efficacy with concurrent antibiotic administration. Kim et al. demonstrated in a Phase 1b RCT^27^ that phage therapy can significantly decrease the concentration of *E. coli* recovered from the bladder in people who were known to be colonized. This work has now progressed to a large phase 2/3 trial for phage therapy in acute UTI. The Zaitsev et al. RCT^33^ reported significant efficacy with bladder instillation of phage in women with recurrent UTI.

Microbiologic cure was reported after approximately two weeks from the start of therapy in most cases, and clinical improvement was monitored via symptom score questionnaires. However, not all of the RCTs show efficacy. An RCT using phage therapy for men with complicated UTI or recurrent uncomplicated UTI^51^ demonstrated that placebo was superior to a phage therapy cocktail with *in vitro* efficacy against their isolates.

This was attributed to a therapeutic benefit from bladder irrigation regardless of lack of active treatment. Interestingly, the phage therapy cocktail was non-inferior to antibiotic standard of care, although both performed poorly as compared with placebo. Regardless of the efficacy results, none of the trials reported significant adverse events.

Understanding the safety considerations of phage therapies is crucial for their responsible and sustainable use. Collection of safety information ensures that phage-based therapies are effective and compliant with regulatory standards. In terms of safety, only 5.4% (3/55) of the included studies reported adverse events after phage administration. It is essential to carefully consider missing data and develop strategies to address any gaps in reporting, since 60% of the articles failed to specify adverse events.

From this review, we conclude that in most cases, phages were a successful treatment option and could potentially be used instead of, or as an adjuvant therapy with antibiotics. Bacteriophage therapy was effective both when a single-phage and a multi-phage cocktail was administered, although most studies failed to provide insight into potential phage resistance or associated adverse events. In conclusion, phages have been demonstrated to be effective in treating infections with negligible safety concerns, in agreement with other recent systematic reviews on phage therapy.^17,83–85^

### Limitations

This systematic review has several limitations. First, our systematic review had high heterogeneity. We assessed results from many different types of UTIs and included articles from all years, languages, and animal systems. Further, we chose to retain studies in our analyses even if our assessment deemed them to be only marginally acceptable. Our comprehensive, historical approach was intentional, as this field has not been studied in detail.

However, this heterogeneity presented some challenges in drawing conclusions on the safety and efficacy of phage therapy and identifying whether there may be an optimal treatment strategy. Without consistent information presented in each study, we were restricted to descriptive analysis rather than statistical or meta-analyses. Despite these limitations, there was a promising trend for the success of phage therapy in treating UTIs. It should be acknowledged that investigators tend to publish only positive results (publication bias), which could affect the type and number of phage therapy studies available for analysis.

Moreover, systematic reviews without language restrictions have the inherent risk of translation and interpretation errors that may have impacted our analyses. To mitigate this effect, we used translation software that was recently validated for its high accuracy.^86^ We feel the benefit of adding these data outweighs any challenges related to translation and interpretations.

In addition, The Joanna Briggs Institute cautions against using absolute scores for quality assessment of case series.^87^ Therefore, we did not provide a numeric score for the studies. Due to the historical and comprehensive nature of this review, we elected to keep all results, regardless of their overall appraisal status.

Our team was multidisciplinary and composed of individuals with different levels of education, training, and expertise. Three of the authors are phage scientists, two are physicians, and one is an experienced reference librarian. One co-primary reviewer was consistent (P.B.H.) throughout the process, whereas the second co-primary reviewer changed three times (A.M.A., G.J.G., and J.D.C.) due to operational factors.

This may have led to inconsistencies. We attempted to limit this impact by ensuring each reviewer had training on our protocol before reviewing articles. Pilot studies were also done to ensure consistency and, our adjudicator, a phage scientist (A.P.H.), resolved the conflicts between various independent reviewers for each round of the article review process, except for the initial round of title and abstract screening and the critical appraisal (G.J.G.).

### Future directions

Our findings suggest that phage therapy is safe and effective in treating UTIs in a variety of animal models and humans. Although there has been a recent increase in the number of studies using phage therapy for treating UTIs, the quality of these studies is variable. Thus, there is a need for researchers and clinicians to use standardized treatment, monitoring, and reporting protocols and, ideally, to share this information with a phage registry.

Recently, a process called the Standardised Treatment and Monitoring Protocol (STAMP) was approved in Australia for patients receiving phage therapy.^88^ The Phage Australia group anticipates that results from clinical trials following the STAMP protocol will be more homogenous and provide stronger evidence of the safety and efficacy of phage therapy. We foresee that future systematic reviews on phage therapy for UTIs will have less heterogeneity, making quantitative analyses possible.

As far as we know, this is the largest systematic review for any single clinical indication treated with phage therapy. There were four other systematic reviews for different clinical sequelae, bone and joint infections (*n* = 20 and *n* = 17), superficial bacterial infections (*n* = 27), and chronically infected wounds infections (*n* = 13).^83,89–91^ We identified two other systematic reviews that evaluated phage therapy across multiple clinical syndromes, including UTIs.

Uttebroek et al.'s review included only human-based studies published in English after 2000.^17^ Meanwhile, Gómez-Ochoa et al. employed a broader approach where they searched for animal-only studies published in any language, but their date range was limited to database inception, which was 1963.^84^

Our lack of date, language, and research subject restrictions allowed us to capture relevant literature dating back to 1926 across four different languages since the use of phages for therapy predates the antibiotic era.^92^ We recommend that systematic reviews evaluating phage therapy for other syndromes implement a similar search strategy encompassing all years and all languages to provide a global perspective in the field. This is particularly important, as more than 20% of the studies in our review were published in languages other than English.

We observed that phage therapy for UTIs in humans historically has a high success rate rivaling antibiotics of today, with few if any adverse events reported. We anticipate that the safety and efficacy demonstrated in our review may be useful in guiding the development of phage therapy clinical practice guidelines, treatment regimens, and dosing schedules. In addition, this review may serve as useful evidence supporting policymaking and research grant applications regarding phage therapy.

## Conclusion

In this comprehensive systematic review, we analyzed reports on the safety and efficacy of phage therapy in UTIs and suggest that phage therapy can be a promising approach to treating UTIs caused by antibiotic-resistant bacteria. However, even recent studies have limitations, including small sample sizes, lack of randomized controlled trials, and limited data on the long-term effects of phage therapy.

Thus, larger well-designed clinical trials are warranted to establish the clinical effectiveness and safety of phage therapy for UTIs. While this review supports the potential of phage therapy as a valuable intervention for the treatment of UTIs, more research is needed to establish the long-term safety and efficacy of the therapy and to support its broader use.

## Supplementary Material

Supplemental data

Supplemental data

Supplemental data

Supplemental data

Supplemental data

## References

[1] Zeng Z, Zhan J, Zhang K, et al. Global, regional, and national burden of urinary tract infections from 1990 to 2019: An analysis of the global burden of disease study 2019. World J Urol 2022;40(3):755–763. 35066637 10.1007/s00345-021-03913-0

[2] Alos JI. Epidemiology and etiology of urinary tract infections in the community. Antimicrobial susceptibility of the main pathogens and clinical significance of resistance. Enferm Infecc Microbiol Clin 2005;23 Suppl 4:3–8. 16854352 10.1157/13091442

[3] Yang X, Chen H, Zheng Y, et al. Disease burden and long-term trends of urinary tract infections: A worldwide report. Front Public Health 2022;10:888205. 35968451 10.3389/fpubh.2022.888205 PMC9363895

[4] Cooper CJ, Khan Mirzaei M, Nilsson AS. Adapting drug approval pathways for bacteriophage-based therapeutics. Front Microbiol 2016;7:1209. 27536293 10.3389/fmicb.2016.01209 PMC4971087

[5] Chan BK, Abedon ST, Loc-Carrillo C. Phage cocktails and the future of phage therapy. Fut Microbiol 2013;8(6):769–783. 10.2217/fmb.13.47 23701332

[6] Tay WH, Chong KKL, Kline KA. Polymicrobial–host interactions during infection. J Mol Biol 2016;428(17):3355–3371. 27170548 10.1016/j.jmb.2016.05.006

[7] Divya Ganeshan S, Hosseinidoust Z. Phage therapy with a focus on the human microbiota. Antibiotics (Basel) 2019;8(3):1. 10.3390/antibiotics8030131 PMC6783874 31461990

[8] Khalifa L, Brosh Y, Gelman D, et al. Targeting *Enterococcus faecalis* biofilms with phage therapy. Appl Environ Microbiol 2015;81(8):2696–2705. 25662974 10.1128/AEM.00096-15 PMC4375334

[9] Liao KS, Lehman SM, Tweardy DJ, et al. Bacteriophages are synergistic with bacterial interference for the prevention of *Pseudomonas aeruginosa* biofilm formation on urinary catheters. J Appl Microbiol 2012;113(6):1530–1539. 22985454 10.1111/j.1365-2672.2012.05432.x PMC3501575

[10] Gu Y, Xu Y, Xu J, et al. Identification of novel bacteriophage vB_EcoP-EG1 with lytic activity against planktonic and biofilm forms of uropathogenic *Escherichia coli*. Appl Microbiol Biotechnol 2019;103(1):315–326. 30397766 10.1007/s00253-018-9471-x

[11] Mittal S, Sharma M, Chaudhary U. Biofilm and multidrug resistance in uropathogenic *Escherichia coli*. Pathog Glob Health 2015;109(1):26–29. 25605466 10.1179/2047773215Y.0000000001 PMC4445292

[12] Fernebro J. Fighting bacterial infections-future treatment options. Drug Resist Updat 2011;14(2):125–139. 21367651 10.1016/j.drup.2011.02.001

[13] Verbeken G, Huys I, Pirnay JP, et al. Taking bacteriophage therapy seriously: A moral argument. Biomed Res Int 2014;2014:621316. 24868534 10.1155/2014/621316 PMC4020481

[14] Leitner L, Kessler TM, Klumpp J. Bacteriophages: A panacea in neuro-urology? Eur Urol Focus 2020;6(3):518–521. 31732462 10.1016/j.euf.2019.10.018

[15] Malik S, Sidhu PK, Rana J, Nehra K. Managing urinary tract infections through phage therapy: A novel approach. Folia Microbiol 2020;65:217–231. 31494814 10.1007/s12223-019-00750-y

[16] Shiryaev A, Vasilyev A, Zaitsev A, et al. Prospects of using bacteriophages in urological practice. Urologiia 2019;15(6):131–136. 32003183

[17] Uyttebroek S, Chen B, Onsea J, et al. Safety and efficacy of phage therapy in difficult-to-treat infections: A systematic review. Lancet Infect Dis 2022;22(8):e208–e220. 35248167 10.1016/S1473-3099(21)00612-5

[18] Żaczek M, Weber-Dąbrowska B, Międzybrodzki R, et al. Phage prevalence in the human urinary tract—Current knowledge and therapeutic implications. Microorganisms 2020;8(11):1802. 33212807 10.3390/microorganisms8111802 PMC7696197

[19] Higgins J, Thomas J, Chandler J, et al. Cochrane Handbook for Systematic Reviews of Interventions Version 6.2, 2021. Cochrane: Canada; 2021.

[19a] Rethlefsen ML, Kirtley S, Waffenschmidt S, et al. PRISMA-S: an extension to the PRISMA statement for reporting literature searches in systematic reviews. Syst Rev 2021;10(1):1–9. 33499930 10.1186/s13643-020-01542-z PMC7839230

[20] McGowan J, Sampson M, Salzwedel DM, et al. PRESS peer review of electronic search strategies: 2015 guideline statement. J Clin Epidemiol 2016;75:40–46. 27005575 10.1016/j.jclinepi.2016.01.021

[21] Wang S, Scells H, Clark J, et al. (eds.) From little Things Big Things Grow: A Collection with Seed Studies for Medical Systematic Review Literature Search. Proceedings of the 45th International ACM SIGIR Conference on Research and Development in Information Retrieval; 2022, pp. 3176–3186.

[22] Clark J, Glasziou P, Del Mar C, et al. A full systematic review was completed in 2weeks using automation tools: A case study. J Clin Epidemiol 2020;121:81–90. 32004673 10.1016/j.jclinepi.2020.01.008

[23] Balk EM, Chung M, Chen ML, et al. Data extraction from machine-translated versus original language randomized trial reports: A comparative study. Syst Rev 2013;2:97. 24199894 10.1186/2046-4053-2-97 PMC4226266

[24] Moola S, Munn Z, Tufanaru C, et al. Chapter 7: Systematic Reviews of Etiology and Risk. Joanna Briggs Institute Reviewer's Manual. The Joanna Briggs Institute; 2017; 17(5):217–269.

[25] Elvers KT, Wilson VJ, Hammond A, et al. Antibiotic-induced changes in the human gut microbiota for the most commonly prescribed antibiotics in primary care in the UK: A systematic review. BMJ Open 2020;10(9):e035677. 10.1136/bmjopen-2019-035677 PMC7507860 32958481

[26] Nadelson S, Nadelson LS. Evidence-based practice article reviews using CASP tools: A method for teaching EBP. Worldviews Evid Based Nurs 2014;11(5):344–346. 25156640 10.1111/wvn.12059

[27] Kim P, Sanchez A, Kime J, et al. Phase 1b Results of pharmacokinetics, pharmacodynamics, and safety for LBPEC01, a CRISPR-Cas3 enhanced bacteriophage cocktail targeting Escherichia coli that cause urinary tract infections. Open Forum Infect Dis 2021;8(SUPPL 1):S633.

[28] Bertoye A, Courtieu AL. Treatment of infections caused by pyocyanic bacilli with bacteriophages adapted by selection. J Méd Lyon 1960;41:739–751. 13800030

[29] Schultz EW. Bacteriophage as a therapeutic agent in genito-urinary infections: Part II. Cal West Med 1932;36(2):91–96. 18742054 PMC1658124

[30] Slopek S, Durlakowa I, Weber-Dabrowska B, et al. Results of bacteriophage treatment of suppurative bacterial infections. I. General evaluation of the results. Arch Immunol Ther Exp (Warsz) 1983;31(3):267–291. 6651484

[31] Gainey AB, Daniels R, Burch A-K, et al. Recurrent ESBL *Escherichia coli* urosepsis in a pediatric renal transplant patient treated with antibiotics and bacteriophage therapy. Pediatr Infect Dis J 2023;42(1):43–46. 36201671 10.1097/INF.0000000000003735

[32] Terwilliger A, Clark J, Karris M, et al. Phage therapy related microbial succession associated with successful clinical outcome for a recurrent urinary tract infection. Viruses. 2021;13(10):2049. 34696479 10.3390/v13102049 PMC8541385

[33] Zaitsev AV, Arefeva OA, Sazonova NA, et al. Results of a clinical study of the drug efficacy and safety for intravesical administration based on bacteriophages in therapy in patients with chronic recurrent cystitis. Gynecology 2021;23(6):578–585.

[34] Abed SY, Alsakini AH, Mohammad MK, et al. A novel broad-host-range phage for treatment of mouse model of *Escherichia coli* urinary tract infection. Trop J Nat Prod Res 2022;6(4):488–493.

[35] Ali BM, Gatea Kaabi SA, Al-Bayati MA, et al. A novel phage cocktail therapy of the urinary tract infection in a Mouse Model. Arch Razi Inst 2021;76(5):1229–1236. 35355758 10.22092/ari.2021.356004.1762 PMC8934106

[36] Aslanov B, Dolgiy A. Bacteriophages for treating urinary tract infections. Int J Infect Dis 2020;101(Supplement 1):113.

[37] Bao H, Zhou Y, Shahin K, et al. The complete genome of lytic Salmonella phage vB_SenM-PA13076 and therapeutic potency in the treatment of lethal *Salmonella Enteritidis* infections in mice. Microbiol Res 2020;237:126471. 32298944 10.1016/j.micres.2020.126471

[38] Bao J, Wu N, Zeng Y, et al. Non-active antibiotic and bacteriophage synergism to successfully treat recurrent urinary tract infection caused by extensively drug-resistant *Klebsiella pneumoniae*. Emerg Microb Infect 2020;9(1):771–774. 10.1080/22221751.2020.1747950 PMC7170350 32212918

[39] Caldwell JA. Bacteriophagy in urinary infections following the administration of the bacteriophage therapeutically. Arch Intern Med 1928;41(2):189–197.

[40] Chen Y, Sun E, Yang L, et al. Therapeutic application of bacteriophage PHB02 and its putative depolymerase against *Pasteurella multocida* capsular type A in mice. Front Microbiol 2018;9(AUG):1678. 30131774 10.3389/fmicb.2018.01678 PMC6090149

[41] Corbellino M, Kieffer N, Kutateladze M, et al. Eradication of a multidrug-resistant, carbapenemase-producing *klebsiella pneumoniae* isolate following oral and intra-rectal therapy with a custom made, lytic bacteriophage preparation. Clin Infect Dis 2020;70(9):1998–2001. 31414123 10.1093/cid/ciz782

[42] Deng LY, Yang ZC, Gong YL, et al. Therapeutic effect of phages on extensively drug-resistant *Acinetobacter baumannii*-induced sepsis in mice. Zhonghua Shao Shang Za Zhi 2016;32(9):523–528. 27647067 10.3760/cma.j.issn.1009-2587.2016.09.003

[43] Dufour N, Clermont O, La Combe B, et al. Bacteriophage LM33_P1, a fast-acting weapon against the pandemic ST131-O25b:H4 *Escherichia coli* clonal complex. J Antimicrob Chemother 2016;71(11):3072–3080. 27387322 10.1093/jac/dkw253

[44] Gordillo Altamirano FL, Kostoulias X, Subedi D, et al. Phage-antibiotic combination is a superior treatment against *Acinetobacter baumannii* in a preclinical study. EBioMed 2022;80:104045. 10.1016/j.ebiom.2022.104045 PMC9097682 35537278

[45] Govorov A, Shiryaev A, Vasilyev A, et al. P0136 Do bacteriophages really prevent urinary tract infections? Eur Urol 2021;79(Supplement 1):S196.

[46] Johri AV, Johri P, Hoyle N, et al. Case report: Chronic bacterial prostatitis treated with phage therapy after multiple failed antibiotic treatments. Front Pharmacol 2021;12:692614. 34177601 10.3389/fphar.2021.692614 PMC8222915

[47] Khawaldeh A, Morales S, Dillon B, et al. Bacteriophage therapy for refractory *Pseudomonas aeruginosa* urinary tract infection. J Med Microbiol 2011;60(Pt 11):1697–1700. 21737541 10.1099/jmm.0.029744-0

[48] Dolgolikova A, Huberskaya M, Efimov D, et al. Potential treatment method of recurrent urinary tract infection in postmenopausal kidney transplant recipients: The results of pilot study. Nephrol Dial Transpl 2020;35(SUPPL 3): gfaa142-P1742.

[49] Kuipers S, Ruth MM, Mientjes M, et al. A Dutch case report of successful treatment of chronic relapsing urinary tract infection with bacteriophages in a renal transplant patient. Antimicrob Agents Chemother 2019;64(1):e01281-19. 31611357 10.1128/AAC.01281-19 PMC7187595

[50] Larkum NW. Bacteriophagy in urinary infection part I. The incidence of bacteriophage and of *Bacillus coli* susceptible to dissolution by the bacteriophage in urines. Presentation of cases of renal infection in which bacteriophage was used therapeutically. J Bacteriol 1926;12(3):203–223. 16559210 10.1128/jb.12.3.203-223.1926 PMC374895

[51] Leitner L, Ujmajuridze A, Chanishvili N, et al. Intravesical bacteriophages for treating urinary tract infections in patients undergoing transurethral resection of the prostate: A randomised, placebo-controlled, double-blind clinical trial. Lancet Infect Dis 2020;21(3):427–436. 32949500 10.1016/S1473-3099(20)30330-3

[52] Leszczynski P, Weber-Dabrowska B, Kohutnicka M, et al. Successful eradication of methicillin-resistant *Staphylococcus aureus* (MRSA) intestinal carrier status in a healthcare worker—Case report. Folia Microbiol 2006;51(3):236–238. 17004656 10.1007/BF02932128

[53] Letkiewicz S, Lusiak-Szelachowska M, Miedzybrodzki R, et al. Low immunogenicity of intravesical phage therapy for urogenitary tract infections. Antibiotics (Basel). 2021;10(6):627. 34070276 10.3390/antibiotics10060627 PMC8225094

[54] Letkiewicz S, Miedzvbrodzki R, Ktak M, et al. Pathogen eradication by phage therapy in patients with chronic bacterial prostatitis. Eur Urol Suppl 2010;9(2):140.

[55] Letkiewicz S, Miedzybrodzki R, Fortuna W, et al. Eradication of Enterococcus faecalis by phage therapy in chronic bacterial prostatitis—Case report. Folia Microbiol 2009;54(5):457–461. 19937220 10.1007/s12223-009-0064-z

[56] Luo X, Liao G, Liu C, et al. Characterization of bacteriophage HN48 and its protective effects in Nile tilapia Oreochromis niloticus against *Streptococcus agalactiae* infections. J Fish Dis 2018;41(10):1477–1484. 30117534 10.1111/jfd.12838

[57] Manolov DG, Kosturkov GB. The effect of dysentery bacteriophage on the course of experimental cystitis produced in guinea-pigs by *Shigella flexneri*. J Hyg Epidemiol Microbiol Immunol 1965;9(4):455–459. 5893861

[58] Nishikawa H, Yasuda M, Uchiyama J, et al. T-even-related bacteriophages as candidates for treatment of *Escherichia coli* urinary tract infections. Arch Virol 2008;153(3):507–515. 18188500 10.1007/s00705-007-0031-4

[59] Pagava KI, Metskhvarishvili GJ, Korineli IA, et al. [Per os given bacteriophages changes the clinical course of diseases caused by bacterial agents in children]. Georgian Med News 2012(211):60–66. 23131986

[60] Pasricha CL, deMonte AJH. Bact. Alkalescens in infection of the urinary tract and bacteriophage therapy. Ind Med Gaz 1941;76(7):414–416. 29013649 PMC5185163

[61] Perepanova TS, Darbeeva OS, Kotliarova GA, et al. [The efficacy of bacteriophage preparations in treating inflammatory urologic diseases]. Effektivnost’ preparatov bakteriofagov pri lechenii vospalitel'nykh urologicheskikh zabolevanii. Urol Nefrol (Mosk) 1995(5):14–17. 8571474

[62] Proskurov VA. [Specific therapy in staphylococcal urethritis]. Spetsificheskaia terapiia pri stafilokokkovykh uretritakh. Vestn Dermatol Venerol 1973;47(5):83–84. 4271160

[63] Pushkar DY, Zaitsev AV, Arefieva OA, et al. Bacteriophages in recurrent urinary tract infection in patients with painful bladder syndrome/interstitial cystitis. Clinical cases. Urologiia 2022(3):115–118.

[64] Qin J, Wu N, Bao J, et al. Heterogeneous *Klebsiella pneumoniae* co-infections complicate personalized bacteriophage therapy. Front Cell Infect Microbiol 2021;10:608402. 33569355 10.3389/fcimb.2020.608402 PMC7868542

[65] Rostkowska OM, Miedzybrodzki R, Miszewska-Szyszkowska D, et al. Treatment of recurrent urinary tract infections in a 60-year-old kidney transplant recipient. The use of phage therapy. Transplant Infect Dis 2020;23(1):e13391. 10.1111/tid.13391 32599666

[66] Slopek S, Durlakowa I, Weber-Dabrowska B, et al. Results of bacteriophage treatment of suppurative bacterial infections. II. Detailed evaluation of the results. Arch Immunol Ther Exp (Warz) 1983;31(3):293–327. 6651485

[67] Slopek S, Durlakowa I, Weber-Dabrowska B, et al. Results of bacteriophage treatment of suppurative bacterial infections. III. Detailed evaluation of the results obtained in further 150 cases. Arch Immunol Ther Exp (Warz) 1984;32(3):317–335. 6395825

[68] Slopek S, Kucharewiczkrukowska A, Weberdabrowska B, et al. Results of bacteriophage treatment of suppurative bacterial-infections. 4. Evaluation of the results obtained in 370 cases. Arch Immunol Ther Exp (Warz) 1985;33(2):219–240. 2935115

[69] Slopek S, Kucharewiczkrukowska A, Weberdabrowska B, et al. Results of bacteriophage treatment of suppurative bacterial-infections. 5. Evaluation of the results obtained in children. Arch Immunol Ther Exp (Warz) 1985;33(2):241–259. 2935116

[70] Slopek S, Kucharewiczkrukowska A, Weberdabrowska B, et al. Results of bacteriophage treatment of suppurative bacterial-infections. 6. Analysis of treatment of suppurative *staphylococcal* infections. Arch Immunol Ther Exp (Warz) 1985;33(2):261–273. 2935117

[71] Slopek S, Weberdabrowska B, Dabrowski M, et al. Results of bacteriophage treatment of suppurative bacterial-infections in the years 1981–1986. Arch Immunol Ther Exp (Warz) 1987;35(5):569–583. 3455647

[72] Tothova L, Celec P, Babickova J, et al. Phage therapy of *Cronobacter*-induced urinary tract infection in mice. Med Sci Monitor 2011;17(7):BR173–BR178. 10.12659/MSM.>16271 PMC3539570 21709627

[73] Ujmajuridze A, Chanishvili N, Goderdzishvili M, et al. Adapted bacteriophages for treating urinary tract infections. Front Microbiol 2018;9:1832. 30131795 10.3389/fmicb.2018.01832 PMC6090023

[74] Ujmajuridze A, Jvania G, Chanishvili N, et al. Phage therapy for the treatment for urinary tract infection: Results of in-vitro screenings and in-vivo application using commercially available bacteriophage cocktails. Eur Urol Suppl 2016;15(3):e265.

[75] Vasilyev AO, Sazonova NA, Melnikov VD, et al. The experience of using a bacteriophages-based complex antibacterial and analgesic drug in gel formulation in women who underwent various instrumental and diagnostic and treatment interventions. Gynecology 2020;22(3):42–48.

[76] Weber-Dabrowska B, Dabrowski M, et al. Studies on bacteriophage penetration in patients subjected to phage therapy. Arch Immunol Ther Exp (Warz) 1987;35(5):563–568. 3332066

[77] Wehrbein HL, Nerb L. Bacteriophage in the treatment of urinary infections. With an appendix on the technique of phage preparation. Am J Surg 1935;29(1):48–53.

[78] Zaldastanishvili E, Leshkasheli L, Dadiani M et al. Phage therapy experience at the Eliava Phage Therapy Center: Three cases of bacterial persistence. Viruses. 2021;13(10):1901. 34696331 10.3390/v13101901 PMC8540005

[79] Zeng Y, Bao J, Tan D, et al. Application of phage in patients with urinary tract pandrug-resistant *Klebsiella pneumoniae* infection. Chin J Urol 2020;41(9):677–680.

[80] Zilisteanu C, Ionescu H, Ionescu-Dorohoi T, et al. [Treatment of urinary infections with bacteriophage-autovaccine-antibiotics]. Considerations sur le traitement des infections urinaires par l'association bacteriophage-autovaccin-antibiotiques. Arch Roum Pathol Exp Microbiol 1971;30(2):195–207. 5146295

[81] Зоpкин C, Шaхновский Д. Possibilities of bacteriophage therapy in the treatment of patients with complicated urinary tract infection [in Russian]. Pediatric Pharmacology 2013;10(4):132–138.

[82] Altamirano FLG, Kostoulias X, Subedi D, et al. Phage-antibiotic combination is a superior treatment against *Acinetobacter baumannii* in a preclinical study. EBioMedicine 2022;80:104045. 35537278 10.1016/j.ebiom.2022.104045 PMC9097682

[83] Clarke AL, De Soir S, Jones JD. The safety and efficacy of phage therapy for bone and joint infections: A systematic review. Antibiotics 2020;9(11):795. 33182795 10.3390/antibiotics9110795 PMC7697170

[84] Gomez-Ochoa SA, Pitton M, Valente LG, et al. Efficacy of phage therapy in preclinical models of bacterial infection: A systematic review and meta-analysis. Lancet Microbe 2022;3(12):e956–e968. 36370748 10.1016/S2666-5247(22)00288-9

[85] Miedzybrodzki R, Borysowski J, Weber-Dabrowska B, et al. Clinical aspects of phage therapy. Adv Virus Res 2012;83:73–121. 22748809 10.1016/B978-0-12-394438-2.00003-7

[86] Jackson JL, Kuriyama A, Anton A, et al. The Accuracy of google translate for abstracting data from Non-English-Language Trials for Systematic Reviews. Ann Intern Med 2019;171(9):677–679. 31357212 10.7326/M19-0891

[87] Munn Z, Barker TH, Moola S, et al. Methodological quality of case series studies: An introduction to the JBI critical appraisal tool. JBI Evid Synth 2020;18(10):2127–2133. 33038125 10.11124/JBISRIR-D-19-00099

[88] Khatami A, Foley DA, Warner MS, et al. Standardised treatment and monitoring protocol to assess safety and tolerability of bacteriophage therapy for adult and paediatric patients (STAMP study): Protocol for an open-label, single-arm trial. BMJ Open 2022;12(12):e065401. 10.1136/bmjopen-2022-065401 PMC9743374 36600337

[89] Duplessis CA, Biswas B. A review of topical phage therapy for chronically infected wounds and preparations for a randomized adaptive clinical trial evaluating topical phage therapy in chronically infected diabetic foot ulcers. Antibiotics 2020;9(7):377. 32635429 10.3390/antibiotics9070377 PMC7400337

[90] Genevière J, McCallin S, Huttner A, et al. A systematic review of phage therapy applied to bone and joint infections: An analysis of success rates, treatment modalities and safety. EFORT Open Rev 2021;6(12):1148. 35003759 10.1302/2058-5241.6.210073 PMC8722473

[91] Steele A, Stacey HJ, De Soir S, et al. The safety and efficacy of phage therapy for superficial bacterial infections: A systematic review. Antibiotics 2020;9(11):754. 33138253 10.3390/antibiotics9110754 PMC7692203

[92] Chanishvili N. Phage therapy—History from Twort and d'Herelle through Soviet experience to current approaches. Adv Virus Res 2012;83:3–40. 22748807 10.1016/B978-0-12-394438-2.00001-3

